# Towards a unified theory of false memory for similar episodes

**DOI:** 10.3758/s13423-025-02855-7

**Published:** 2026-02-19

**Authors:** Colleen M. Parks, Andrew P. Yonelinas, Christopher N. Wahlheim

**Affiliations:** 1 Department of Psychology, University of Nevada, Las Vegas, NV USA; 2 Department of Psychology, University of California, Davis, CA USA; 3 Department of Psychology, University of North Carolina at Greensboro, Greensboro, NC USA

**Keywords:** Process dissociation, Mnemonic similarity, Familiarity and recollection, False memory

## Abstract

Important advances have been made in understanding the roles that recollection and familiarity, true and false memory, as well as pattern separation and pattern completion, play in episodic memory, using experimental paradigms that examine memory for lures that are similar – but not identical – to prior episodes. These include the Process Dissociation Procedure, the Deese-Roediger-McDermott paradigm, and the Mnemonic Similarity Task. However, research using each of these paradigms has remained largely isolated, and a coherent theoretical integration is lacking. In this perspective paper, we argue that memory for similar lures in all these paradigms can be understood within a unified theory in which memory performance reflects the operation of three distinct processes: false recollection, false familiarity, and recollection rejection. Our proposed approach leverages the theoretical insights provided by examining recognition confidence ratings and analyzing false memory receiver operating characteristics. Using extant data from paradigms across all three literatures that collected recognition confidence judgments, we show that this analytic approach can be used to measure each process. Our findings bridge the existing literatures by showing that these three memory processes are functionally distinct and that they underlie performance in each experimental paradigm. This new approach overcomes key limitations of the earlier methods by separating common memory and decision processes across paradigms, and it provides new opportunities to further understand why factors such as healthy aging disrupt the ability to discriminate between similar episodes.

**Supplementary Information:**

The online version contains supplementary material available at 10.3758/s13423-025-02855-7.

## Introduction

We often need to use memory to discriminate between very similar items and past episodes. For example, we may need to know whether the black suitcase in baggage claim is the one we had checked earlier in the day or someone else’s, or whether we took our medication today or yesterday. When our memory fails to provide us with answers to these types of questions, or when it provides us with incorrect or false information, it can have serious consequences, such as taking the wrong luggage or failing to take a critical medication.

Memory researchers have studied the processes underlying the ability to distinguish between similar memories using experimental paradigms that require subjects to discriminate among exact repetitions of studied items, entirely novel items, and, most critically, items that share some, but not all, features with studied items, henceforth referred to as *similar lures*. Three influential experimental paradigms that include similar lures have been developed and used profitably to measure and characterize several core memory mechanisms. The Process Dissociation Procedure (PDP, Jacoby, 1991) has been used to separate the role of recollection and familiarity-based memory responses, the Deese-Roediger-McDermott paradigm (DRM, Roediger & McDermott, 1995) has provided important insights into the nature of true and false memories, and the Mnemonic Similarity Task (MST, Kirwan & Stark, 2007) has been used to outline the roles of hippocampal pattern separation and pattern completion in detecting and rejecting similar lures. We argue that there are striking similarities across these paradigms, but that the literatures in which they are embedded have remained quite separate. As we describe below, similar patterns of findings across these paradigms have been interpreted in entirely different ways. Thus, the field is in a state analogous to the parable of the blind men and the elephant: Each person has a different theory of what an elephant is because they are exploring different parts of the creature. Our challenge, then, is to assemble a broader picture of the common processes underlying these paradigms.

We address this challenge by proposing a unified theory of false memory for similar episodes that leverages a common analytic approach to obtaining process estimates. We propose that memory for similar lures can be understood as reflecting a mixture of three processes: *false recollection*, *false familiarity*, and *recollection rejection*. That is, a similar lure can be incorrectly identified as having been studied if it elicits the retrieval of qualitative information linking the item to the study event. Alternatively, it can be accepted as old if it is perceived as being sufficiently familiar. In addition, however, a lure can be rejected if it elicits the retrieval of information that distinguishes the similar lure from studied items. After describing these paradigms and the processes in more detail, we assess the general utility of this approach by applying it to results from published studies from each of these paradigms. Critically, we show that the mere addition of confidence judgments to recognition paradigms with similar lures allows one to plot “false-memory receiver operating characteristics” (fROCs) and to then derive estimates of these processes for comparison across related paradigms. Finally, we show how this approach overcomes limitations of the earlier approaches, and we consider the implications of the theory and confidence-based measurement method for future studies of episodic memory.

### Recognition paradigms that examine memory for similar lures

**Fig. 1:**
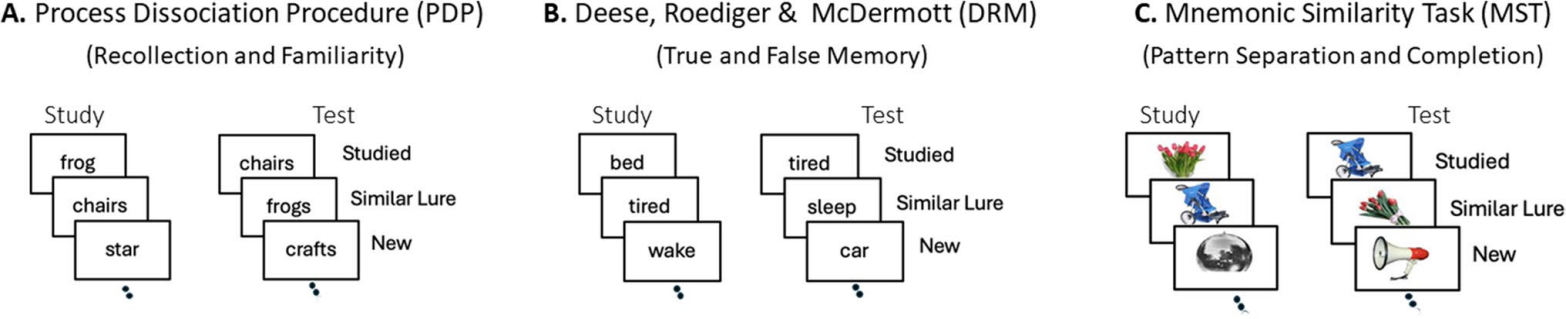
Three common recognition memory paradigms assessing memory for similar lures. **A.** The Process Dissociation Procedure (PDP) measures recollection and familiarity. **B.** The Deese-Roediger-McDermott (DRM) paradigm measures true and false memory. **C.** The Mnemonic Similarity Task (MST) measures hippocampal pattern separation and pattern completion. In each paradigm, subjects study a list of items, followed by a recognition test that contains a random mixture of studied items, similar lures, and nonstudied (new) items. In all three tests, subjects are instructed to respond “old” to studied items, and “new” to nonstudied items; in the MST, a “similar” response is typically included as well. In the PDP and DRM paradigms the similar lures should receive a “new” response, whereas in the MST they should receive a “similar” response.

Figure 1 illustrates the three major recognition memory paradigms that include similar lures. In each case, subjects are first presented with a series of study items, and after a delay are given a recognition test that requires them to discriminate between studied items, nonstudied (new) items, and lures that are similar to one or more of the studied items.

#### Process Dissociation Procedure (PDP)

The PDP has been used to separate the contribution of accurate *recollection*, which is the conscious retrieval of qualitative information about a study event, such as what the studied item looked like or where it occurred, from *familiarity*, which reflects an undifferentiated sense of oldness in the absence of recollected details (Jacoby, 1991, 1998) for reviews and critiques see (Dobbins, 2015; Kunnari et al., 2020; Yonelinas & Jacoby, 2012; Zhang et al., 2020). Recollection is critical in tests of source and associative recognition, whereas familiarity can support performance on tests of item recognition (for review, see Yonelinas, 2002). In the PDP, recollection is measured as the ability to discriminate between studied items and similar lures (i.e., recollection should lead one to accept an item that was previously presented as having been studied but to reject a similar lure as having not been studied). In contrast, familiarity should not support this discrimination but rather should lead both studied items and similar lures to be more familiar than entirely new items.

One example of a PDP experiment is the *plurality test* illustrated in Fig. 1A, in which subjects are told to respond “old” to studied words that are in the same plurality as in the study phase, and “new” to both plurality-reversed lures and entirely new items (Hintzman & Curran, 1994; Rotello et al., 2000). Other common approaches include *source tests* that require subjects to discriminate between items from two different study lists (e.g., Jacoby, 1991; Yonelinas, 1994; also see Johnson et al., 1993), *synonym tests* that require subjects to discriminate between studied words and synonyms of studied words (Lampinen & Arnal, 2009), *object orientation tests* where subjects must discriminate between studied objects and objects studied in a different spatial orientation (Lampinen et al., 2006), and *associative tests* where subjects discriminate between intact and rearranged word pairings (Rotello et al., 2000).

Numerous studies have used the PDP to determine the functional nature of recollection and familiarity (for reviews, see Diana et al., 2006; Yonelinas et al., 2024; Yonelinas & Jacoby, 2012), as well as to identify their neural substrates and determine the state of these processes in different populations (de Chastelaine et al., 2023; Friehs et al., 2021; Köhler & Martin, 2020; Kwon et al., 2023; Zhang et al., 2020). For example, one topic that has been extensively examined using the PDP, as well as in studies using the DRM and MST paradigms, is the effect of aging. Numerous studies using the PDP have shown that healthy aging is associated with a gradual decrease in recollection while familiarity remains largely unaffected (for review, see Koen & Yonelinas, 2014). These deficits are due in part to reductions in hippocampal integrity, which is a structure that is critical in forming novel associations needed for recollection (Duarte et al., 2008; Schoemaker et al., 2014, 2017; Yonelinas & Parks, 2007).

#### Deese-Roediger-McDermott (DRM) paradigm

The DRM paradigm reliably induces false memories in the lab and so has formed one of the foundations of our understanding of true and false memory (Gallo et al., 2006; Roediger & Gallo, 2022; Roediger & McDermott, 1995). In the DRM paradigm (see Fig. 1B), subjects study lists of associated words, which are also associated with a critical nonstudied lure (e.g., *bed*, *tired*, *wake*, etc., are associated with the critical lure *sleep*). At test, subjects receive a recognition test that includes studied and entirely new items as well as critical (similar) lures. Subjects falsely recognize the similar lures as having been studied, even when they are instructed to avoid falsely accepting lures, and in some conditions, they are as likely to recognize the similar lure (i.e., a false memory) as they are to recognize the studied items (i.e., true memories).

The DRM paradigm has revealed that false memories can be quite frequent and that they are often associated with highly confident and vivid recollections (Abichou et al., 2022; Norman & Schacter, 1997; Roediger & McDermott, 1995), which can arise because of spreading activation from studied to lure items during encoding, and failures to adequately monitor the products of retrieval (for a review, see Gallo, 2010). The procedure has also been used to uncover the neural systems that support true and false memories (e.g., (Friehs et al., 2021; H. Kim & Cabeza, 2007), and to examine how true and false memory vary across populations. For example, healthy older adults have been found to be particularly susceptible to false memories (for a review, see Devitt & Schacter, 2016).

#### Mnemonic Similarity Task (MST)

The MST was designed to separate hippocampal *pattern separation* from other memory processes such as *pattern completion* (see Fig. 1C; for a review, see Stark et al., 2019). Pattern separation is a process whereby similar experiences or stimuli are encoded as distinct, non-overlapping representations. It is thought to be particularly well supported by the dentate gyrus (DG) subfield of the hippocampus because the DG exhibits sparse patterns of firing and neurogenesis, and is capable of forming highly distinctive representations for individual events (Leutgeb et al., 2007; Yassa & Stark, 2011). In contrast, pattern completion reflects a more general memory function whereby a partial retrieval cue leads to the retrieval (i.e., completion) of missing event details (Guzowski et al., 2004; Horner et al., 2015).

In the MST, subjects typically study pictures of objects and are then presented with a recognition test that includes pictures of studied objects, new objects, and similar lure objects (i.e., an object that is slightly different from a studied item in some way such as in color, orientation, etc.). Subjects are typically required to indicate if each item was old, similar, or new. The ability to identify a similar lure as similar, rather than old or new, is attributed, in part, to pattern separation computations in the DG. In contrast, the ability to discriminate between old and new items reflects more general retrieval processes supported, in part, by pattern completion. It is generally acknowledged that the MST does not provide a process pure measure of hippocampal pattern separation (i.e., other cognitive processes and brain regions can also support lure rejections; for a review, see Stark et al., 2019). This limitation notwithstanding, the paradigm has been used to identify factors that promote rejections of similar lures (Loiotile & Courtney, 2015; Sifuentes Ortega & Peigneux, 2024), examine increased failures to reject lures in vulnerable populations, and investigate the neural mechanisms underlying such rejections (for a review, see Stark et al., 2019). For example, DG lesions are associated with pronounced deficits in lure discrimination performance thought to reflect pattern separation more than pattern completion (Baker et al., 2016), and healthy aging is also associated with a similar pattern of impairments (e.g., Stark et al., 2013; Stark & Stark, 2017).

#### Comparing the similar-lure paradigms

These three paradigms are similar in the sense that they require subjects to discriminate between studied items, similar lures, and new items, but they can differ with respect to the specific responses they require and the stimulus materials that are used. For example, in the PDP and DRM paradigms, subjects are often given two response options (old/new) and are instructed to respond “old” to studied items and “new” to similar lures and nonstudied items, whereas in the MST, subjects are often given three response options (old/similar/new), which allows subjects to specifically label similar items as “similar.” However, comparable response options can be used across paradigms. For example, in the PDP subjects can be asked to make old/new discriminations as well as to further indicate if the item was from a critical study source or from a similar but different study source (e.g., Yonelinas, 1999). Moreover, in all three paradigms subjects are sometimes required to provide old/new confidence ratings or remember/know/new responses for each test item (Kim & Yassa, 2013; Loiotile & Courtney, 2015; Stark et al., 2015; Szőllősi et al., 2020; Wahlheim et al., 2024). Another potentially important difference across paradigms is that PDP and DRM studies have typically examined memory for words, whereas those using the MST have most often used pictures of objects. However, this is not a necessary difference as both words and pictures have been used in each paradigm (Děchtěrenko et al., 2021; Lampinen et al., 2006; Ly et al., 2013; Wang et al., 2019). Finally, whereas in the DRM the lures are typically semantically or associatively related to the targets (e.g., “sleep” is semantically related to “tired”), in the MST they are usually perceptually and semantically related to the targets (e.g., different tulip bouquets look alike and they feature the same type of flower). However, a number of DRM studies have also examined the effects of perceptual similarity (e.g., Coane et al., 2021), and in the PDP, the targets and lures can be either semantically related (e.g., in synonym discrimination tests) or perceptually and semantically related (e.g., in object-orientation discrimination tests). So, the types of responses and stimulus materials used in individual experiments can vary, but these factors do not reflect *essential* differences across the three paradigms. However, in the *Discussion*, we consider how differences in response options and materials can affect the mnemonic processes underlying decisions about similar lures.

One unique aspect of the MST is that, in some versions of the task, the degree of target-lure similarity varies across items (see Lacy et al., 2010). This has provided important insights into mechanisms of lure discrimination in populations with varying hippocampal function (Davidson et al., 2019; Das et al., 2020; Stark et al., 2013; Trelle et al., 2021). For example, age-related differences (in healthy aging or mild cognitive impairment) are typically larger for intermediate levels of similarity than for the highest and lowest levels, which has been taken as evidence for representational rigidity in the dentate gyrus/CA3 network (Stark et al., 2013). According to this view, the need for greater degrees of distinction between lures and targets reflects a shift away from pattern separation and toward pattern completion, due to reduced input from the entorhinal cortex and hyperactivity in CA3. The three-process model we describe below could potentially provide converging evidence for this shift by further characterizing the retrieval processes contributing to lure discrimination across variations in lure similarity. Unfortunately, no studies have reported confidence responses to lures that are grouped according to different levels of similarity, so future studies will be needed to evaluate this claim.

Despite the obvious similarities across these paradigms, they are used to assess fundamentally different memory constructs (i.e., recollection/familiarity, true/false memory, and pattern separation/completion), and they lead to different conclusions about various aspects of memory function. As one example, consider what these approaches have told us about the effects of heathy aging on memory. According to the PDP, healthy aging leads to a deficit in recollection that does not impact familiarity; according to the DRM, aging leads individuals to more often falsely recognize events that never occurred; and according to the MST, aging leads to a particularly pronounced deficit in pattern separation and bias towards pattern completion. But do findings from these three paradigms really tell us about fundamentally different memory processes? We think not, and instead argue that a unified account can explain the results across all three paradigms. Although each of these approaches reasonably approximates what they were designed to measure, they are all incomplete because they only focus on part of the broader picture. Specifically, they emphasize only one or two processes that underlie performance on similar lures tests, whereas we argue that at least three separable processes underlie performance.

## A unified theory of false memory for similar episodes: False recollection, false familiarity, and recollection rejection

**Fig. 2:**
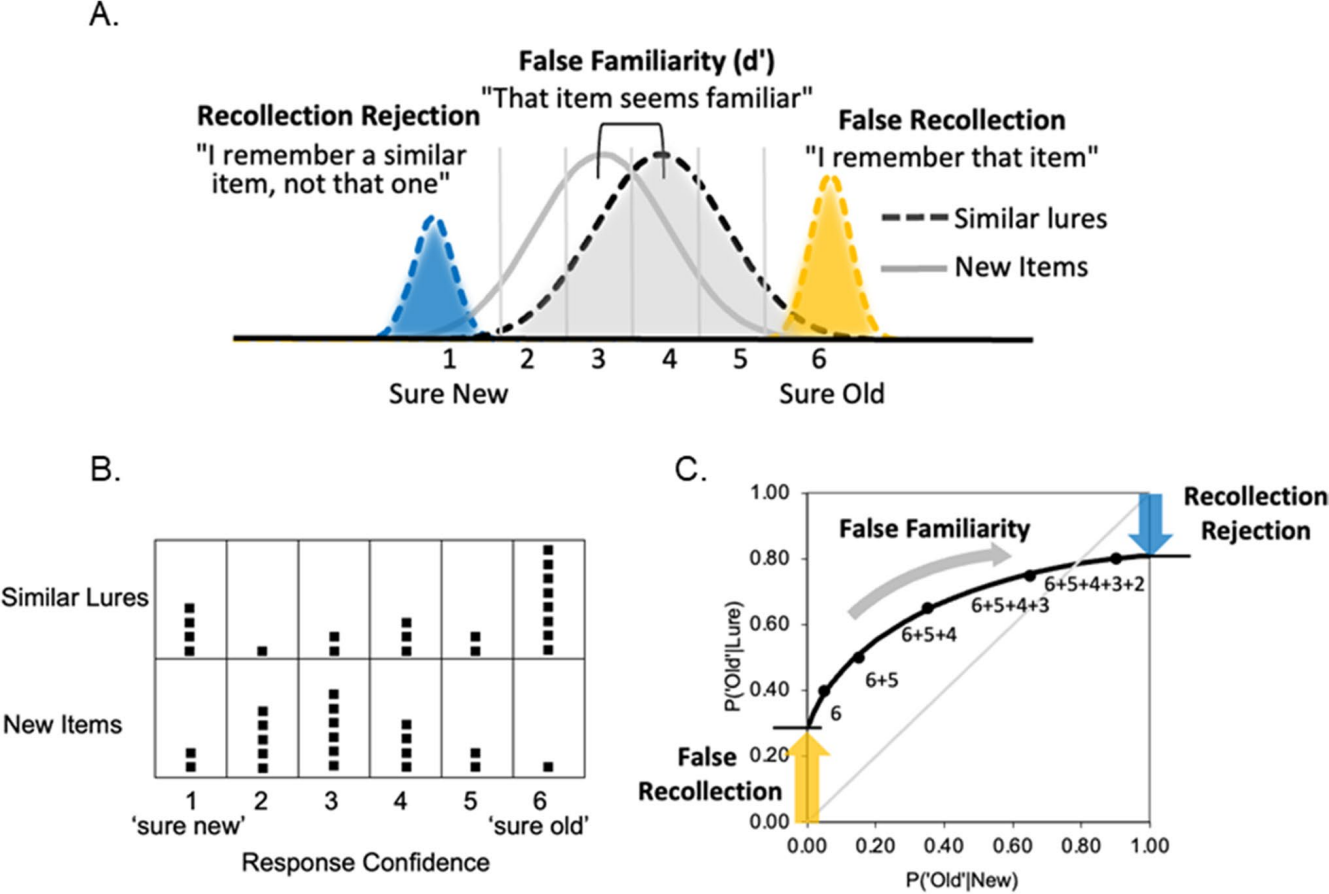
**A.** A three-component mixture model of recognition memory judgments for similar lures. Memory strength/confidence distributions are shown for false familiarity (gray), recollection rejection (blue), and false recollection (yellow). **B.** Hypothetical distributions of confidence responses that might be observed for similar lures and new items (each square represents a single response). **C.** False-memory receiver operating characteristics (fROCs) based on recognition confidence ratings along with the fitted mixture model function. The left-most point of the fROC reflects high confidence old trials (i.e., 6 responses), and each subsequent point includes the next most confident trials (i.e., 6 + 5 responses, etc.). The mixture model is fit to the observed points using a nonlinear regression method and is shown as the continuous solid curve. The projected left intercept of the function provides an estimate of false recollection, whereas the right intercept of the function provides an estimate of recollection rejection, and the curvilinearity of the observed function reflects the extent to which familiarity contributes to performance (i.e., as the familiarity of the lures increases, the familiarity distributions move farther apart, and the fROC will become more curvilinear).

We propose a three-component model (see Fig. 2A) that builds on and unifies the theoretical foundations of the existing similar-lure recognition memory paradigms. The approach assumes that similar lures can result in *false recollection*, whereby subjects retrieve qualitative information erroneously linking the test item to the earlier study item; *false familiarity* whereby subjects accept a test item on the basis that it is perceived as sufficiently familiar; and *recollection rejection* whereby subjects recollect qualitative information about the study item that allows them to correctly reject the test item as being different from what was studied. The three processes are conceptualized within a mixture signal detection framework that allows for variations in memory confidence and response criteria. The model is based on the dual-process signal-detection model of item recognition (Yonelinas, 1994), and on the phantom recollection model (Lampinen et al., 2006), which incorporates false recollection into the dual-process model (for related cognitive and neurocomputational models of these processes, see Bastin, et al., 2019; Diana, et al., 2007; Elfman et al., 2008; Norman & O’Reilly, 2003; Sherman et al., 2003).

The model assumes that similar lures will be more familiar than new items because lure features are more likely to match what has been stored in memory  during the study phase than the features of entirely new items. The perceived familiarity is expected to vary from one item to the next, resulting in two Gaussian familiarity strength distributions, as assumed by signal detection theories of memory (i.e., the gray distributions in Fig. 2A; e.g., Murdock, 1965). Lures that are sufficiently familiar will be accepted as old, and greater familiarity strength is assumed to lead to higher levels of recognition confidence. False familiarity can therefore be measured as a *d’* value that is the average difference in familiarity strength between lures and new items.

The model also assumes that recognition judgments for similar lures will be affected by recollection of episodic details of studied items, which will influence performance in two ways. First, subjects can use recollection to reject a similar lure if they can recollect a criterial detail (or details) of a corresponding studied item that is similar but not identical to that of the test item (e.g., “I saw *frog*, not *frogs*” or “I saw words related to *sleep*, but I did not see *sleep*” or “I saw a picture of *tulips*, but in a different arrangement”), resulting in the blue distribution in Fig. 2A. Second, subjects can falsely recollect a similar lure as a studied item if they recollect details linking the lure to the study item but fail to recollect any criterial details that can be used to reject the item as having been different for the study event (e.g., “I saw *frogs*” or “I saw *sleep*” or “I saw those *tulips*”) resulting in the yellow distribution in Fig. 2A. PDP and DRM studies have shown that recollection supports high confidence recognition judgments (Roediger & McDermott, 1995; Yonelinas, 1999); we therefore assume that recollection rejection will be associated with high confidence “new” responses, and false recollection will be associated with high confidence “old” responses. For example, on a 6-point confidence scale (Sure New = 1 to 6 = Sure Old) recollection rejection will lead to a 1 response whereas false recollection would lead to a 6 response. In this way, recollection rejection and false recollection can be measured as probabilities, whereas false familiarity is measured as a *d’* value (but for a discussion of conditions in which the high confidence recollection assumption may be relaxed, see Sherman et al., 2003; Yonelinas & Parks, 2007).

If this model is correct, then recognition confidence judgments will be highly constrained. For example, if one compares the probability of recognizing a lure item with the probability of recognizing a new item across levels of response confidence (e.g., see hypothetical distribution of confidence responses in Fig. 2B), this produces an fROC like the function in Fig. 2C. The model produces a left intercept (reflecting false recollection) and a right intercept (reflecting recollection rejection), and it is curved (reflecting false familiarity). If, on the one hand, these three processes do contribute to lure classification performance, then the fROCs should exhibit all three of these features. In addition, these aspects of the function should be dissociable via selective effects of experimental manipulations on particular processes. If, on the other hand, a simpler two-process approach is viable, then two of these factors may behave in similar ways, and so could be collapsed (e.g., false recollection may just be the opposite of recollection rejection). Moreover, if the model can adequately describe the fROCs, then one can measure the relative roles of these three processes by fitting the model to confidence data. This is analogous to conducting a linear regression to estimate the slope and intercept of a line, but in this case, the model introduces a nonlinear component (i.e., *d’*) to account for false familiarity. Box 1 provides more details on how fROCs can be used to derive process estimates and describes a process calculator in the form of an excel spreadsheet that is available on the Open Science Framework (OSF; https://osf.io/39rs6/).

So, how can we determine if findings in the available literature on recognition of similar lures is consistent with this three-component model? Fortunately, several published studies have included confidence judgments in these paradigms, which allows us to test predictions from our mixture model and derive estimates of the three component processes across the different paradigms. Although a comprehensive test of our approach will require future studies that include assessments of individual subject data, the post hoc analyses of group data that we report below provides a useful proof of concept.

**Box 1** Generating false-memory ROCs and estimating false recollection, false familiarity, and recollection rejection

**Table:** 

ROCs are typically derived from confidence judgments made on a 6-point scale (i.e., 1 = sure new … 6 = sure old), or from old/new judgments followed by 3-point confidence judgments (i.e., low, medium, or high). To ensure stable ROCs that cover a sufficient range of confidence values, subjects are typically instructed to use the entire scale, and at least 60 lure and 60 new trials are collected from each subject (see Hautus et al., 2021; Yonelinas & Parks, 2007). Confidence judgments can then be used to measure the processes underlying performance by fitting a mixture model to the observed confidence data. For example, a modifiable spreadsheet is available on the OSF (https://osf.io/39rs6/overview) that fits the model to the observed fROC data points by minimizing the sum of squared errors for each observed point in the x- and y-dimensions between the observed and predicted fROCs. A spreadsheet for fitting standard item recognition ROCs is available at https://yonelinas.faculty.ucdavis.edu/roc-analysis/. Similar estimates can be derived using other methods such as maximum log likelihood methods (Koen et al., 2017). In brief, the number of observed confidence responses for similar lures and new items are used to plot the observed fROC (i.e., the proportion of similar lures and new items accepted as old as a function of response confidence; also see Fig. 2), and the mixture model is fit to the observed data to find the model parameters that provide the best fit to the observed data. The model assumes that a similar lure will be accepted as old if it is falsely recollected (R_f_), or in the absence of false recollection and recollection rejection (R_r_), the familiarity of the lure exceeds the response criterion (i.e., F_lure_ > c, which according to signal detection theory is the area under the lure item distribution that exceeds the response criterion (c) given the distance between the new and lure distributions is d’: Φ(*d’*/2-c)). So, P(‘old’|lure) = R_f_ + (1- R_f_ - R_r_) * Φ(*d’*/2-c). Moreover, a new item is assumed to be accepted as old if the familiarity of the new item exceeds the response criterion (i.e., F_new_ > c, or Φ(-*d’*/2-c)). So, P(‘old’|new) = Φ(-*d’*/2-c). The two equations can be combined to define the ROC: P(‘old’|lure) = P(‘old’|new) + R_f_ + (1- R_f_ - R_r_) * (Φ(*d’*/2-c)) – Φ(-*d’*/2-c)	

### Receiver operating characteristic studies of similar lure recognition

**Fig. 3:**
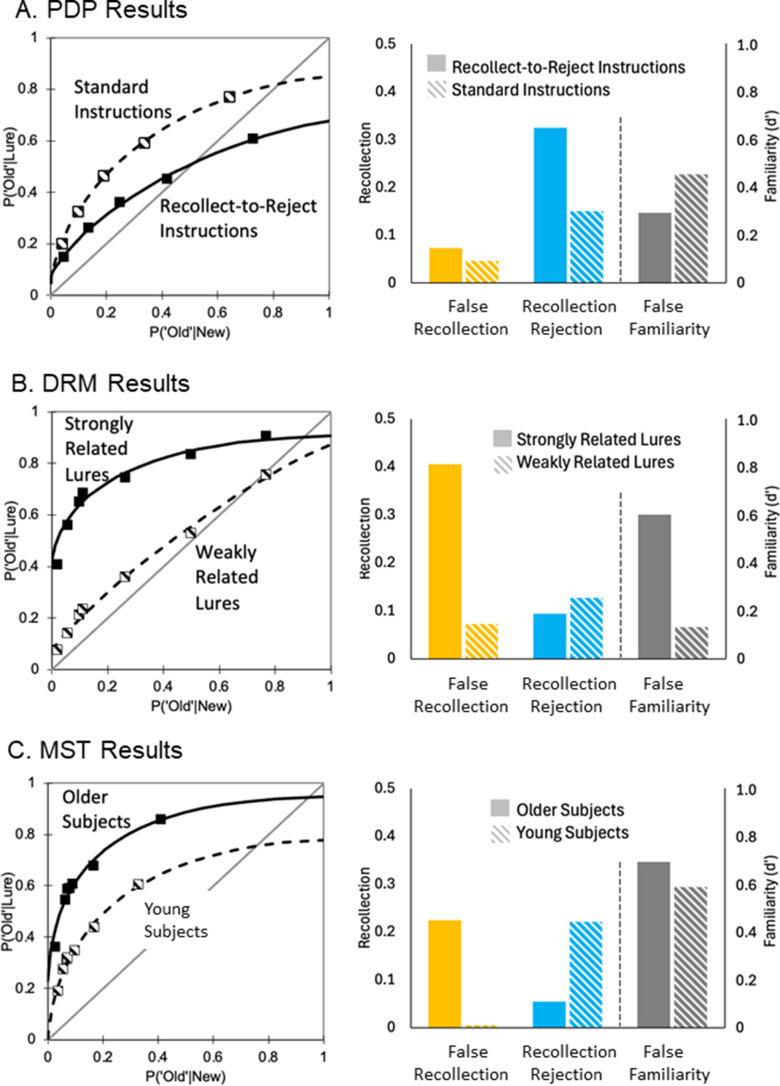
Observed false-memory receiver operating characteristics (fROCs; squares in left panels), fit by the model (solid and dashed lines in left panels), and model-derived parameter estimates (right panels). **A.** Results from a Process Dissociation Procedure (PDP) study (Rotello et al., 2000) examining recognition for plurality reversed lures under standard instructions or when instructed to focus on a recollect-to-reject strategy. **B.** Results from a Deese-Roediger-McDermott paradigm (DRM) study examining recognition for lures that were either strongly or weakly semantically related to the studied items (Gatti et al., 2024). **C.** Results from a Mnemonic Similarity Task (MST) study comparing similar lure recognition for older and younger subjects (Stark et al., 2015). In each paradigm, the model function fit the fROC points well. In addition, the parameter estimates indicated that instructing subjects to use a recollect-to-reject strategy selectively increased recollection rejection (A); conversely, strongly compared to weakly related lures led to higher false recollection and false familiarity estimates (B); whereas older adults showed higher false recollection and lower recollection rejection estimates than young adults while exhibiting similar levels of false familiarity (C).

**Fig. 4:**
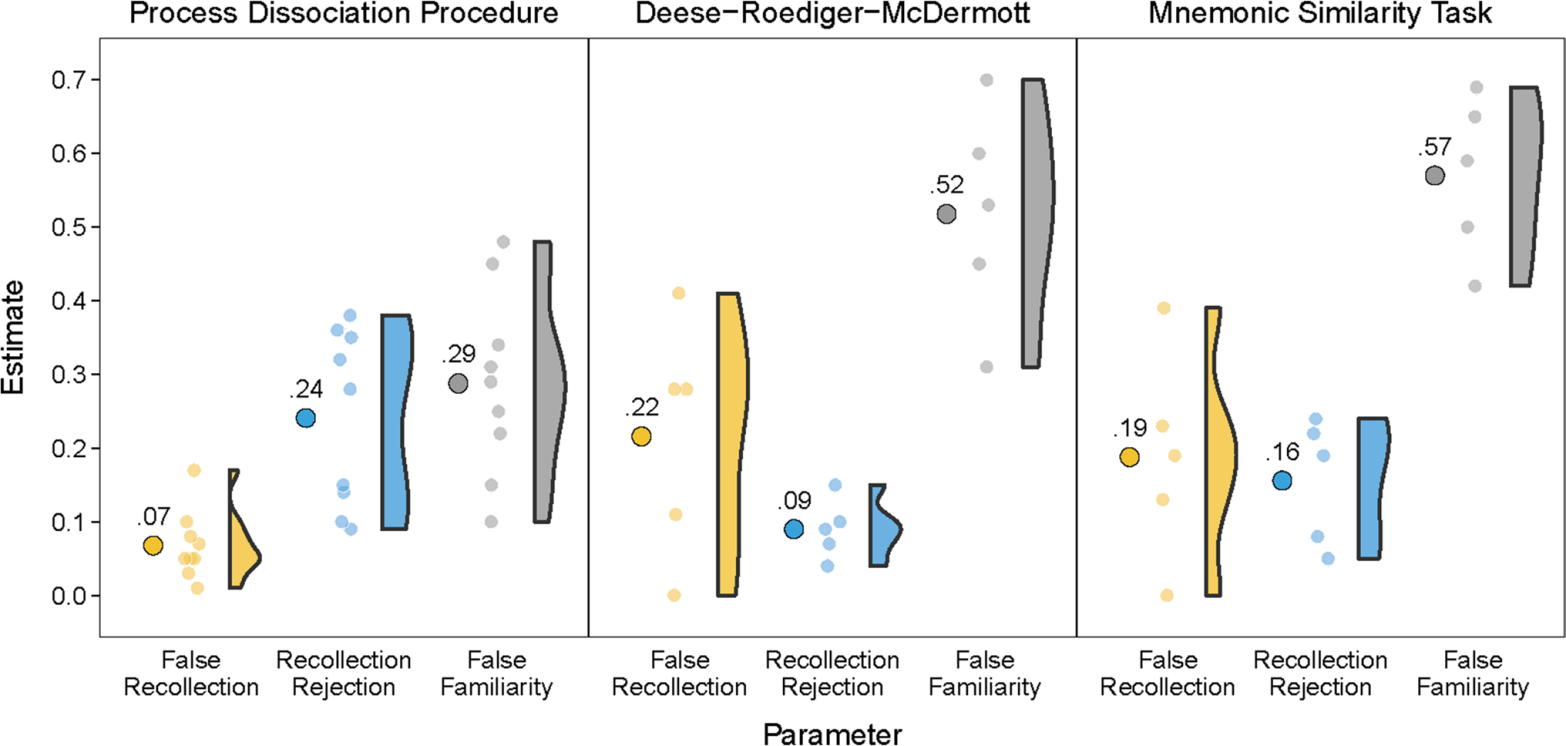
Parameter estimates of false recollection, recollection rejection, and false familiarity in all studies that using old/new confidence tests in the Process Dissociation Procedure, Deese-Roediger-McDermott, and Mnemonic Similarity Task paradigms. Larger circles indicate the average estimates across studies, smaller circles indicate the estimates from individual studies, and half violins indicate the distributions of estimates across studies.

We identified 11 published studies that reported recognition confidence judgments for similar lures, several of which included multiple experimental conditions, resulting in 20 fROCs. In each case, we fit the model to the observed functions to assess whether the model was consistent with the data and to derive parameter estimates. Figure 3 illustrates the observed fROCs, the estimated model functions, and the parameter estimates from three illustrative experiments, one from each of the similar-lure paradigms. The fROCs from all the other available data sets are presented in the Appendix (Fig. A1) in the Electronic Supplementary Material (ESM), and Fig. 4 shows the parameter estimates in each condition from each of the available studies.

#### Assessing fROC shape

The observed fROC points in each of the three paradigms were fit well by the mixture model (Fig. 3, left panels). For example, Fig. 3A shows that in a plurality-reversal PDP experiment (Rotello et al., 2000), the model fit the observed points almost perfectly under standard instructions (i.e., “accept studied items and reject all others”) and under instructions to use a recollect-to-reject strategy (i.e., “pay particular attention to the plurality of each word”). Similarly good fits were observed in studies using other PDP variants (see Appendix, Fig. A1, panel A (ESM)), including studies examining “list membership” (Yonelinas, 1994, across short and long lists and after one and three study repetitions), memory for whether word-pairs were rearranged or intact (Rotello et al., 2000), and memory for whether object pictures appeared in the same or altered orientations (Lampinen et al., 2006, under full and divided attention during study).

Similarly, Fig. 3B shows that the model also accurately described the shape of the fROCs in the DRM paradigm when the lures were strongly or weakly related to the studied items (Gatti et al., 2024). Similarly good fits were observed in other DRM studies (see Appendix, Fig. A1, panel B (ESM)), including studies examining memory for negative and neutral words (Yüvrük & Kapucu, 2022), when words were studied under transcranial and sham stimulation conditions (Friehs et al., 2021), and during fMRI scanning (Kim & Cabeza, 2007).

Finally, Fig. 3C indicates that the model also provided a good fit of the fROCs in the MST paradigm. For example, the model fit the fROCs from older and young adults well (Stark et al., 2015, Exp 4). Similarly good fits were observed in other MST studies (see Appendix, Fig. A1, panel C (ESM)), including a similar study examining college-aged subjects (Szőllősi et al., 2020), and another study with studied items presented one and three times (Loiotile & Courtney, 2015). The finding that the model accurately described the shape of the observed fROC data in all three paradigms across various conditions and populations suggests that, despite their differences, the three paradigms reflect the same underlying processes and can be understood within a single theoretical framework.

The accuracy of the model fits was verified by examining the sum of squared errors which was less than .002 in all the studies, indicating that the model accounted for a vast majority of the observed variance in behavior in each study. We also examined item recognition ROCs for old versus new items in these paradigms and found them to be consistent with those observed in the broader item recognition ROC literature (i.e., they were curvilinear and asymmetrical, and fit well by dual-process signal detection and the unequal variance signal detection models; for review see Yonelinas & Parks, 2007). Because these ROC results are already well established, we do not focus on them in the current paper.

#### Assessing the parameter estimates

Figure 3 (right panels) presents the parameter estimates derived from the corresponding fROCs (left panels). In all three experiments, all three process estimates were above zero, suggesting that each process contributed to performance in each paradigm (see Fig. 4 for estimates from all studies). Importantly, the three processes were also functionally distinct. For example, Fig. 3A shows that instructing subjects to use a recollect-to-reject strategy produced a relatively selective increase in the recollection rejection parameter. Conversely, Fig. 3B shows that increasing the similarity between the lures and studied items led to greater false recollection and false familiarity but did not affect recollection rejection. Finally, Fig. 3C shows higher false recollection and lower recollection rejection estimates for older than young adults, whereas age had little or no effect on false familiarity. These functional dissociations show that the three component processes are distinct from one another (i.e., they are differentially sensitive to different experimental manipulations), rather than reflecting different manifestations of the same underlying process (e.g., false recollection is not simply the inverse of recollection rejection). Thus, a simpler approach that includes only two of these underlying processes such as recollection/familiarity, true/false memory, or pattern separation/completion, would likely be insufficient to characterize the processes underlying recognition decisions for similar lures.

Although the same model can account for performance in the three different paradigms, an examination of the process estimates from all studies (see Fig. 4) points to potentially important differences in the extent to which each process contributed to performance in each of the paradigms. For example, PDP studies often led to higher estimates of recollection rejection (blue points) and lower estimates of false familiarity (gray points) than DRM and MST studies. Higher estimates of recollection rejection in the PDP studies could have arisen because subjects are often explicitly instructed to use recollection to reject the similar lures, whereas in the other paradigms the utility of using recollection rejection is often only implied by the test procedures. False familiarity may have been lower in the PDP paradigms because the lures are often not semantically related in the studied items (i.e., in list discrimination studies the lures are unrelated items that were studied in a different experimental context), whereas they are typically semantically and/or perceptually related in the DRM and MST paradigms. In addition, studies using the PDP often showed less false recollection (yellow points) than the studies using the other two paradigms. This may be because in PDP studies the distinction between the studied items and similar lures is made obvious to the subject (e.g., in a plurality judgment test subjects know that the study item was either the singular or the plural version of a word, and so they know which feature of the test item distinguishes between studied items and similar lures). In contrast, in the MST and DRM paradigms there is often no single feature that distinguishes the studied item from similar lures, making it more difficult to avoid false recollection. Future work directly contrasting these three procedures using systematic manipulations will be critical in testing these hypotheses, and in characterizing the conditions under which these processes are most likely to occur.

#### Overcoming limitations of earlier approaches

The proposed model overcomes several key shortcomings of the earlier approaches. For instance, the PDP paradigm was designed to measure recollection and familiarity, but it does not account for false recollection and false familiarity. Our results suggest that false recollection of similar lures does occur in studies using the PDP, and although it happens less often than in the other paradigms, when it occurs, it will bias estimates of recollection and familiarity derived using the traditional methods. The broader implication for the PDP literature is that recollection and familiarity are susceptible to error, and so it is critical to incorporate these two types of false memory when measuring performance and interpreting those results. Future PDP and related source and associative memory studies that include confidence responses will provide more precise measures of recollection and familiarity and further delineate when these processes are accurate or in error.

The traditional DRM paradigm provides an effective way of measuring true and false memory, but there is considerable debate about the processes underlying these false recognition responses (Gallo, 2010). The current results indicate that there are at least three separate retrieval processes underlying false memories. Some false memories reflect false recollection whereby subjects retrieve false episodic information linking the test item to the encoding events, whereas other false memories reflect high levels of perceived familiarity. These conclusions are consistent with previous DRM studies that have found that false memories can be associated with remember responses whereby subjects claim to retrieve qualitative information about the study event (Roediger & McDermott, 1995). The current results, however, highlight that the false recognition of similar lures often arises because some of the critical lures are simply more familiar than novel items (Watson et al., 2003). Moreover, the current results also highlight that recollection rejection occurs in the DRM paradigm in the sense that subjects can use it to avoid falsely accepting similar lures as having been studied (Gallo, 2004). Although false familiarity and recollection rejection have been discussed in some DRM studies (Gallo, 2010), the current approach provides a way of directly measuring each of these processes. Future DRM studies using confidence measures to separate these three processes will advance our understanding of why false memories occur and may even be useful in reducing false memory by facilitating the developing procedures that target each of these critical processes.

In the MST, the ability to discriminate between lures and new items is often used as an index of a DG-based pattern separation process, whereas the ability to discriminate between studied and new items is often used as a measure of pattern completion. It is, however, widely acknowledged that the correct rejection of similar lures is only an indirect assay of DG function, because other neural and cognitive processes can also influence that ability (Kim & Yassa, 2013; Loiotile & Courtney, 2015; Stark et al., 2019; Szőllősi et al., 2020; Wahlheim et al., 2023, 2024). The current results highlight those concerns by indicating that responses to similar lures reflect a mixture of three separate processes, not all of which may be dependent on DG-based pattern separation.

What, then, are the implications of our proposed approach for MST studies that have used a three-response procedure (i.e., “old,” “similar,” or “new”)? The current results show that memory for similar lures reflects the combined effects of three different memory processes. In this way, “similar” responses to similar lures can arise for several different reasons. For example, a “similar” response could arise if subjects use recollection rejection to identify a similar lure as being different from an item that was studied. However, “similar” responses can also arise on the basis of familiarity. For example, similar lures that evoke intermediate levels of familiarity should also lead to a “similar” response (i.e., an item that seems less familiar than most studied items, but more familiar than new items). Although successful pattern separation based on the DG may be critical for the responses based on recollection rejection, it may be less important for the familiarity-based responses. Consequently, “similar” responses will overestimate the role of the hippocampus, even when performance is corrected by false alarms to new items.

For this reason, we think it is essential to measure old/new confidence rather than old/similar/new judgments. However, another potentially useful approach is to combine the two methods. For example, an old/new confidence rating could be followed by an old/similar/new rating for each item. In this way, both methods of analyzing the results could be used, and the confidence responses could be used to separate the “similar” responses that are the most likely to rely heavily on recollection-based pattern separation from familiarity-based pattern separation. That is, a lure that receives a “sure new (1)” response and also receives a “similar” response would likely reflect recollection rejection and so should be reliant on the hippocampus. In contrast, a lure that receives an intermediate level of old/new confidence (e.g., a 4 or a 5 response) followed by a “similar” judgment likely reflects a familiarity-based pattern separation that presumably would be less reliant on hippocampal pattern separation.

The current approach may also be important in furthering our understanding of the neural mechanisms underlying memory for similar lures in all three of the similar lure paradigms. For example, one of the studies that we examined (Kim & Cabeza, 2007) used fMRI with the DRM paradigm and found that high-confidence accurate recognition elicited greater hippocampal activity than high-confidence false recognition, whereas high-confidence false recognition elicited greater activity in a frontoparietal network. This pattern was interpreted as reflecting neural activity underlying recollection and familiarity, respectively. Our fROC analysis of the confidence data from that study is consistent with the claim that the responses to similar lures were based on false familiarity rather than false recollection in the sense that estimates of false familiarity were above chance in that study (*d’* = 0.53), whereas false recollection of the related lures was effectively zero. In addition, high-confidence correct rejections also elicited MTL activity, possibly reflecting recollection rejection, which is consistent with the above zero estimates of recollection rejection (.10). If the current approach is correct, then under conditions in which false recollection is more likely, one would expect to see hippocampal involvement in both high-confidence true and high-confidence false recognition trials. Future neuroimaging and lesion studies using these paradigms with old/new confidence judgments may further elucidate the relationship between these processes, the medial temporal lobes, and larger scale networks.

## Conclusions, limitations, and future directions

A growing body of literature using the PDP, DRM, and MST paradigms to examine our ability to discriminate between similar episodes has provided rich insights into the processes underlying episodic recognition by clarifying the differences between recollection and familiarity-based memory judgments, characterizing conditions that lead to true and false memories, and linking performance to the processes of hippocampal pattern separation and pattern completion. Although these literatures have been largely isolated, and, on the surface, appear to be addressing fundamentally different questions, our analysis suggests that they can all be understood as reflecting the contribution of three separable memory processes: false recollection, false familiarity, and recollection rejection. We presented a parsimonious three-component model that specifies how recognition of similar lures should vary with response confidence and found that it was in good agreement with published data in all three paradigms. The model builds on well-established signal detection principles, and the theoretical insights provided by the existing PDP, DRM, and MST literatures. The results indicated that all three processes contributed to each of these major memory paradigms and showed that these processes were functionally dissociable, in the sense that they were sensitive to different experimental manipulations. Importantly, the new approach overcomes limitations of earlier approaches and creates a way of bridging those different literatures by revealing that they can all be understood as reflecting the same underlying set of episodic memory processes.

We argue that this approach provides a substantial step toward developing a unified theory of false memory for similar events, which will be useful in guiding future research, but several limitations will need to be addressed. One important goal will be to determine whether the proposed model can be applied to other empirical paradigms, such as tests examining subjective reports of remembering and knowing (Tulving, 1985), tests measuring the precision of recollected information (Harlow & Donaldson, 2013; Zhang & Luck, 2008), tests of eyewitness identification (Brewer & Wells, 2011), or misinformation effects (Loftus & Klemfuss, 2024) including studies examining the effects of correcting false news and assessing the contributions of recollection to reject false information (Wahlheim et al., 2025). We suspect that false recollection, false familiarity, and recollection rejection play important roles in a wide variety of meaningful real-life experiences, and so future studies designed to separate these three processes promise to provide deeper insights into these memory processes.

We also need to determine how the model parameters relate to those derived from alternative modelling approaches, such as multinomial models and multidimension signal detection models (Banks, 2000; Lee & Stark, 2023; Rouder et al., 2008). These types of models have been used in conjunction with each of these three similar lure paradigms, but whether they can produce the types of ROCs that are observed in the similar lure paradigms is not yet clear. The challenge for threshold-based theories like multinomial models is producing the curvilinear ROCs that are observed in tests of item recognition (and in all the fROCs we examined here), whereas the challenge for pure signal detection models is producing the relatively linear ROCs that are observed in tests of associative and source memory (e.g., Rotello et al., 2000; Yonelinas 1999; for review, see Yonelinas & Parks, 2007). A related theoretical issue is whether there may be conditions under which recollection supports both high- and lower-confidence responses (Sherman et al., 2003), in which case it may be necessary to include additional distributional assumptions about recollective strength.

We also need additional studies that directly contrast the effects of different types of stimuli (e.g., words, objects, scenes, and narratives), different types of lure similarity (e.g., lures that are perceptually or conceptually similar), and different populations (e.g., older adults, adults with MCI, etc.) because all three factors are likely to affect performance. For example, older adults perform more poorly than young adults when lures are perceptually similar to studied items, but they are much less impaired when the materials are complex scenes (Güsten et al., 2021) or when they involve changes in narrative event details (Delarazan et al., 2023). By including measures of confidence and assessing the underlying processes, future studies could help explain why age-related deficits are more pronounced under certain conditions.

Moreover, the MST results examined here suggested that aging is associated with the disruption of recollection but not familiarity (at least for object stimuli). However, these age-related recollection deficits were found to occur for two different reasons: Older adults were more likely to falsely recollect similar lures, and they were less likely to use recollection rejection to avoid false alarms. It will be important to determine whether these effects are observed with other types of stimuli and test procedures, and whether the changes in false recollection and recollection rejection reflect one or more distinct neural mechanisms. For example, age-related hippocampal atrophy may be associated with greater false recollection, whereas age-related deficits in recollection rejection may be due to diminished frontally mediated executive function and monitoring processes (Dennis et al., 2022; Kramer et al., 2005). In addition, although these kinds of age-related memory deficits can sometimes be ameliorated using behavioral training and pharmacological interventions (Bakker et al., 2012; Hampstead et al., 2020; Jennings & Jacoby, 2003; Verhaeghen et al., 1992), it is unknown which processes are affected. Thus, using the current approach may allow us to tailor remediation strategies and/or pharmacological treatments to more effectively target a person’s specific episodic memory problems.

In sum, many paradigms have been developed to assess the processes underlying false recognition of similar lures, but they have only focused on subsets of the processes underlying performance. However, we propose that the results from all these paradigms can be understood as reflecting the combined effects of false recollection, false familiarity, and recollection rejection. These three processes can be measured by including an old-new confidence judgment in each of these existing paradigms and using a mixture signal detection model to examine these episodic memory processes. Doing so provides us with a broader picture of the core component processes that lead to accurate discriminations and false endorsements of similar episodes.

## Supplementary Information

Below is the link to the electronic supplementary material.

Supplementary file1 (DOCX 103 KB)

## Data Availability

The publication relies on secondary analysis of data previously published by other authors. These summary statistics, other supplementary materials, and the model calculator are posted on the Open Science Framework.
