# Supplementary material for: Towards a unified theory of false memory for similar episodes
Source: Psychon Bull Rev. 2026 Feb 19;33(2):88. doi: 10.3758/s13423-025-02855-7 (PMC12920419; doi:10.3758/s13423-025-02855-7)
Supplement: Supplementary file 1 — Supplementary file1 (DOCX 103 KB) [file 13423_2025_2855_MOESM1_ESM.docx]

**Appendix**


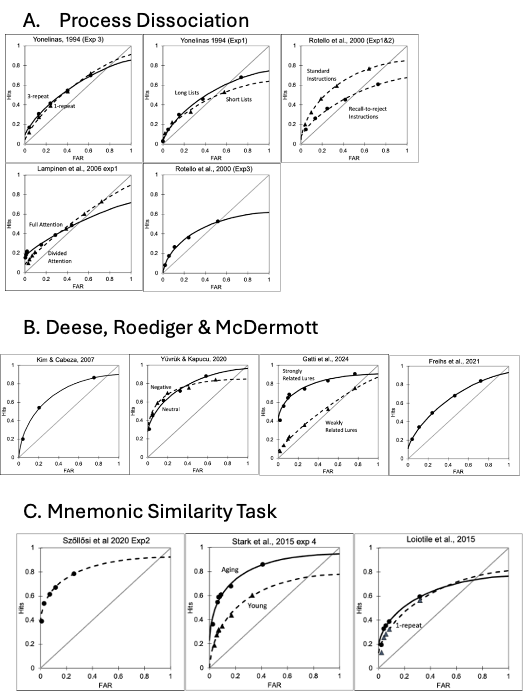


Figure A1. A) Observed ROCs and model fits in studies using the process dissociation procedure. Results are presented from studies using plurality tests in which the lures were plurality reversed (Rotello et al., 2000); source tests in which the lures were from a nontarget study list (Yonelinas, 1994); object orientation tests in which lures were studied objects but from a different spatial orientation (Lampinen et al., 2006); and associative tests in which lures were rearranged word pairs (Rotello et al., 2000). B) Observed ROCs and model fits in studies using the DRM paradigm in which the lures were associated with multiple studied items that were encoded under transcranial stimulation and sham stimulation conditions (Freihs et al., 2021 - anodal and cathodal condition ROCs were similar to the sham and so only the sham ROC is shown); for negative and neutral words (Yüvrük & Kapucu, 2000 - positive words were not different from neutral so are not shown); during fMRI scanning (Kim & Cabeza, 2007); and for lures that were strongly and weakly related to study items (Gatti, Rinaldi, Mazzoni & Vecchi, 2024). C) Observed ROCs and model fits in studies using the MST paradigm, in young and older adults (Stark et al., 2015, Exp 4 – note that results from a second ‘gist’ condition were not examined because subjects were told to treat both old and lure items as old); in college aged subjects (Szollosi, 2020), and for items studied 1 or 3 times (Loiotile, 2015).
